# Downregulation of *KCNMB4* expression and changes in BK channel subtype in hippocampal granule neurons following seizure activity

**DOI:** 10.1371/journal.pone.0188064

**Published:** 2017-11-16

**Authors:** Luke E. Whitmire, Ling Ling, Vladslav Bugay, Chase M. Carver, Santosh Timilsina, Hui-Hsiu Chuang, David B. Jaffe, Mark S. Shapiro, Jose E. Cavazos, Robert Brenner

**Affiliations:** 1 Department of Cell and Integrative Physiology, University of Texas Health Science Center at San Antonio, San Antonio, Texas, United States of America; 2 Department of Biology, University of Texas at San Antonio, San Antonio, Texas, United States of America; 3 Neurology, University of Texas Health Science Center at San Antonio, San Antonio, Texas, United States of America; Tel Aviv University Sackler Faculty of Medicine, ISRAEL

## Abstract

A major challenge is to understand maladaptive changes in ion channels that sets neurons on a course towards epilepsy development. Voltage- and calcium-activated K+ (BK) channels contribute to early spike timing in neurons, and studies indicate that the BK channel plays a pathological role in increasing excitability early after a seizure. Here, we have investigated changes in BK channels and their accessory β4 subunit (*KCNMB4*) in dentate gyrus (DG) granule neurons of the hippocampus, key neurons that regulate excitability of the hippocampus circuit. Two days after pilocarpine-induced seizures, we found that the predominant effect is a downregulation of the β4 accessory subunit mRNA. Consistent with reduced expression, single channel recording and pharmacology indicate a switch in the subtype of channels expressed; from iberiotoxin-resistant, type II BK channels (BK α/β4) that have higher channel open probability and slow gating, to iberiotoxin-sensitive type I channels (BK α alone) with low open probability and faster gating. The switch to a majority of type I channel expression following seizure activity is correlated with a loss of BK channel function on spike threshold while maintaining the channel’s contribution to increased early spike frequency. Using heterozygous β4 knockout mice, we find reduced expression is sufficient to increase seizure sensitivity. We conclude that seizure-induced downregulation of *KCNMB4* is an activity dependent mechanism that increases the excitability of DG neurons. These novel findings indicate that BK channel subtypes are not only defined by cell-specific expression, but can also be plastic depending on the recent history of neuronal excitability.

## Introduction

It is well established that seizures alter the milieu of ion channels and transporters in neurons, some of which are pro-inhibitory and protect against subsequent seizures, whereas others are pro-excitatory and ultimately contribute to epileptogenesis. Changes in ion channels are observed, including inward rectifiers and HCN channels, which provide an adaptive response following seizures [1–5]. However, studies of large conductance voltage- and calcium-activated (BK) potassium channels have suggested that these channels contribute a maladaptive, early response to seizures in cortical neurons [6, 7]. Following a single seizure, blocking BK channels protects against subsequent seizures [6]. Also supporting the concept that BK channels can be pro-excitatory is the finding that humans with a gain-of-function point mutation of the pore-forming α subunit (*Kcnma1*) have epileptic seizures [8]. In addition, action potential (AP) frequency is increased in hypothalamic neurons expressing a gain-of-function BK channel in transgenic mice [9].

One mechanism for reducing the pro-excitatory influence of BK currents is through association with the BK channel accessory β4 subunit. Increased AP frequency is seen in hippocampal granule cells of mice where the inhibitory neuronal β4 (*KCNMB4*) subunit is genetically ablated [10]. This BK channel gain-of-function ultimately results in seizures that are secondarily generalized with a temporal lobe origin [10]. Loss of the β4 subunit also sensitizes neurons to phosphorylation dependent gain-of-function and increases in neuron excitability [11]. As well, the β4 subunit appears to dramatically regulate BK channel surface expression in some cell types [12]. Thus, the regulatory BK channel β4 subunit is well positioned to affect excitability following seizure insults. However the underlying mechanisms and the contributions of the β4 subunit following seizures have not yet been studied.

Here we conduct the first investigation of changes in BK α/β4 subunit expression and function following seizures. We utilize a combination of electrophysiology, RT-qPCR, and pharmacology of BK channels to reveal a downregulaton of β4 and a switch from predominantly α/β4 type II channels to α type I channels within two days following a chemoconvulsant-induced seizure. Current clamp recordings reveal that BK channels normally depolarize the spike threshold, but also facilitate early spike frequency. Following seizures, β4 downregulation is correlated with a loss of BK channel effect on threshold potentials, while the increase of early spike frequency is maintained by BK channels. We conclude that following increased periods of excitability, granule neurons shift from the predominant expression of type II to type I BK channels as a means to increase circuit excitability.

## Materials and methods

The University of Texas Health Science Center at San Antonio Institutional Animal Care and Use Committee (IACUC) approved all experimental procedures involving animals.

### Treatment with pilocarpine and kainic acid

The chemoconvulsants pilocarpine and kainic acid are the most common model for generating temporal lobe epilepsy in rodents [13–15]. Thus we used these compounds to induce seizures and investigate early dentate gyrus changes (2 days post seizure) in the epileptogenesis process. Mice used in this study were C57BL/6J strain, 23 to 28 day old males. *KCNMB4* knockout mice were inbred by 6 generations to the same genetic background. Thirty minutes prior to administration of pilocarpine (200 mg/kg, Tocris Bioscience, Bristol, UK) mice received an injection of scopolamine methylnitrate 1mg/kg (MP Biomedicals, Santa Ana, CA, USA) that does not cross the blood-brain barrier and inhibits peripheral pilocarpine effects [16]. Drugs were delivered via intraperitoneal (I.P.) injection in 0.9% normal saline (pH 7.4). All pilocarpine or kainic acid treated mice used in this study were visually confirmed to showed behavioral signs of seizure activity within two hours following injection, and only mice that scored a 4 (using the Racine scale) were included [17]. A very uniform seizure event was noted with I.P. injection of 200 mg/kg of pilocarpine. Within the first 15 minutes mice would exhibit continuous tremors associated with a rigid tail. Peak seizure activity was observed 60 minutes after injection when mice would display intermittent rearing with upper extremity clonus. Two hours after injection occasional tremors were observed. Following two hours of observation, mice were allowed to recover in home cages with their littermates. Twenty-four hours after seizure activity, the behavior of seizure mice was indistinguishable from untreated mice. Control mice were given an equivalent dose of scopolamine followed by an I.P. injection of vehicle.

A group of five age-matched male mice were used to evaluate the mRNA response to kainic acid. Kainic acid was delivered i.p. at a dose of 10 mg/kg [18]. All five mice had a similar seizure event as the pilocarpine treated group, however initial seizure behavior (tremors and rigid tail) was delayed to 20 minutes and seizure activity lasted to three hours. Mice treated with kainic acid were only used in RT-qPCR analysis 48 hours after injection. Electrophysiology experiments were conducted in pilocarpine or vehicle injected mice that were sacrificed 48 hours after treatment.

### Immunohistology

Mice were deeply anesthetized with isoflurane and transcardially perfused with 25 mL of PBS solution followed by perfusion of 250 mL of 4% paraformaldehyde (PFA) in PBS. Whole brains were dissected and stored in a 20% (weight to volume) solution of sucrose in PBS overnight. The next day frozen coronal sections (50 μm) were cut using a Leica SM 2010R sliding microtome.

#### BK channel immunohistochemistry

**Fig 1:**
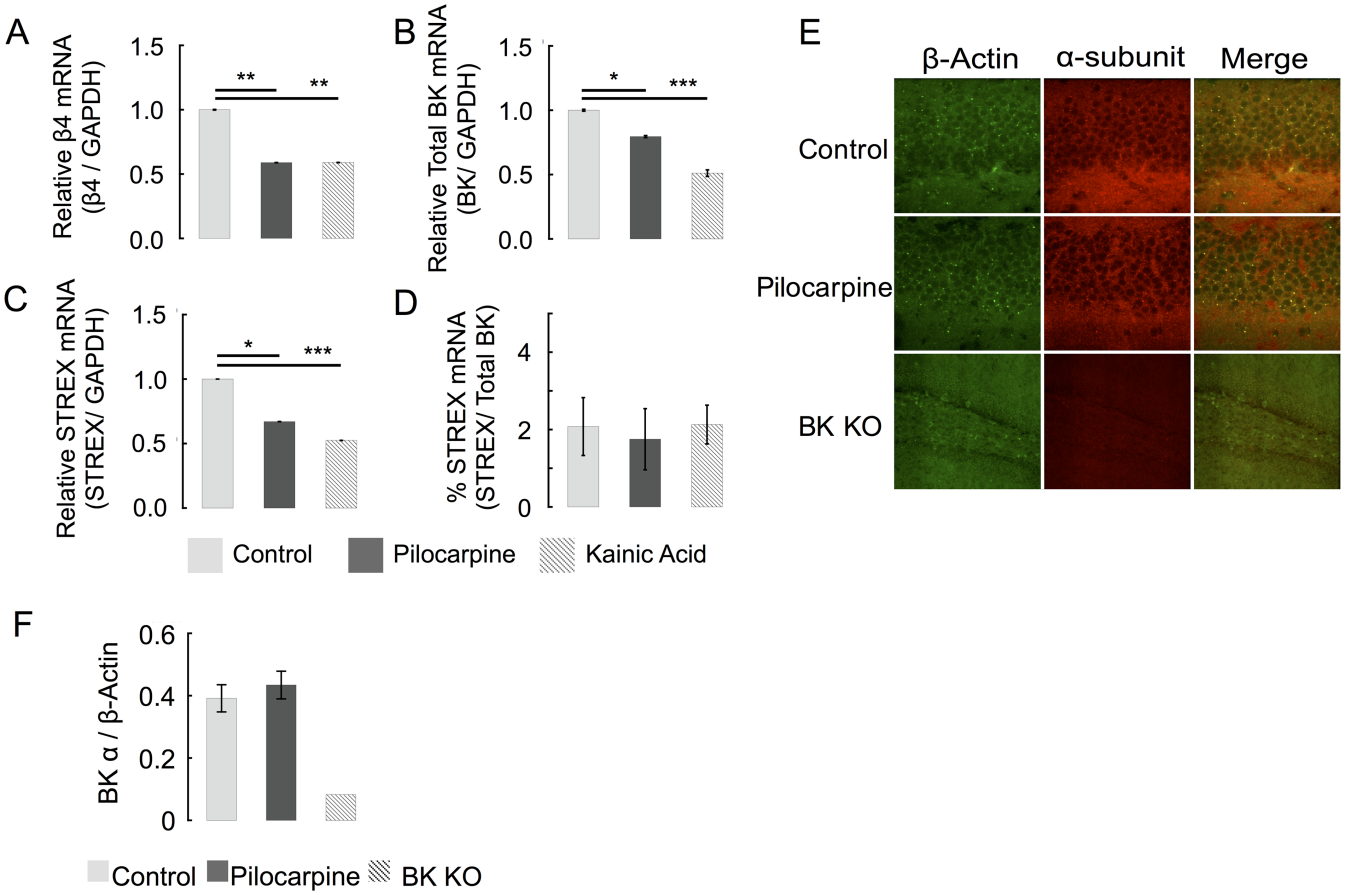
Seizure effect on BK channel subunit expression. (A) RT-PCR of dissected hippocampi shows that seizure activity decreases β4 mRNA expression by 41% ± 0.3. (B) Pilocarpine results in 20% ± 1 less Kcnma1 and kainic acid decrease α-subunit mRNA expression by 49% ± 1. (C) STREX specific primers show a drop in STREX containing mRNA. (D) The relative values of STREX containing and total BK mRNA are unaffected by either seizure model, suggesting that seizure activity does not lead to early regulation of STREX retention in BK channel mRNA. (n = 5 for each group.) (E) Immunoflourescent staining of β-actin (488 nm) and BK α-subunit (555 nm) in dentate gyrus granule cell layer and hilus of hippocampal slices. The wild type sections were taken at a 40X magnification. The knockout sections (bottom row) were taken at a 20X magnification to ensure that there is no staining throughout the section. (F) Average BK α-subunit protein immunofluorescence (normalized to β-actin immunofluorescence) shows no changes 48 hours following pilocarpine. (Control n = 6, Seizure n = 5, and BK α KO n = 2).

Tissue was blocked in PBS-0.1% Tween-20 (PBS-T) containing 4% goat serum (blocking solution) for four hours at room temperature as free-floating sections. Sections were then exposed to primary antibodies in PBS-T with 4% goat serum overnight at 4°C. For total BK channel detection the monoclonal L6/60 anti-BK channel antibody (Antibody Inc, Davis, CA, USA) was used at a 1:1000 dilution in the PBS blocking solution. Specificity of the L6/60 antibody was confirmed by using BK knockout mice (see Fig 1, a kind gift of Dr. Andrea Meredith, University of Maryland). An anti-β-actin antibody (sc-130656, Santa Cruz Biotechnology) was used for normalization at a concentration of 1:2000 in blocking solution. Secondary antibodies included Alexa Fluor 488 goat anti-rabbit IgG and Alexa Flour 555 Goat anti-mouse (Molecular Probes, A-11034 and A-21422), respectively. Secondary antibodies were exposed to sections (after extensive washing in blocking solution) at a concentration of 1:500 for two hours at room temperature. Again, after extensive washing, sections were then mounted on Superfrost Plus slides in Vectashield (Vector Laboratories, Burlingame, CA, USA) and covered with a number one coverslip. Immunostaining was visualized using a Nikon Eclipse FN1 upright microscope using a 60 X Plan Apo objective and swept-field confocal. Nikon NIS Elements (AR 4.12.01) software was used for image capture and analysis. Images were obtained at a fixed exposure rate of 600 ms and the EM gain was set to 300 in a 10 MHz @ 14-bit readout mode. FITC and TRITC excitation were carried out at 80% power of 488 and 561 nm laser emission source, respectively (MIC 400B, Agilent Technologies). BK channel expression is represented as the average ± S.E.M. of the ratio of 488 to 561 signals. Significance between groups was evaluated with an ANOVA.

#### EGFP immunohistochemistry

Heterozygous *KCNMB4* knockout mice were used to study changes in transcription *in situ*. The knockout mice were made by gene-targeting the Clontech EGFP-N1 open reading frame and transcriptional terminator sequence into the first exon of the *KCNMB4* gene [10]. The KCNQ2 EGFP reporter mice were transgenic for a bacterial artificial chromosome (RP23-247P15) containing the Knq2 locus with EGFP targeted to the first exon (GENSAT Cat# 015412). EGFP was detected using a Goat anti-EGFP antibody (1:2000 dilution, Rockland #600-101-215), and Alexa Fluor 488 Donkey anti-goat (1:200, Thermofisher, A11055). MAP2 was detected with Chicken anti-MAP2 (1:5000, Abcam ab5392), and Rhodamine Red, Rabbit anti-chicken (1:100, Jackson Immuno Research, 303-295-008). cFos was detected using a Rabbit anti-cFos (1:10,000, Synaptic Systems, 226003), and Alexa Fluor 568 Donkey anti-Rabbit IgG (1:100, ThermoFisher, A10042). After extensive washing, sections were mounted in anti-fading reagent and imaged on a Nikon Eclipse FN1 upright microscope with a 4x Plan Fluor objective for EGFP and MAP-2 costaining, and with a 20X Plan Apo for the EGFP and cFos costaining. Intensity ratios for EGFP/MAP2 were obtained for each image by altering the laser excitation light source (488 and 561 nm, respectively) at a fixed exposure time of 5 seconds (average of 8 frames) in a 10 MHz @ 14-bit readout mode. EGFP/cFos images were obtained by comparing EGFP fluorescence intensity in cFos-positive granule cells, to a large group of neighboring cells (~10 cells) that were cFos negative.

### RT-qPCR

Hippocampi were dissected in ice-cold cutting solution (described below) and total RNA was collected using a PureLink RNA mini kit (Life Technologies, 12183018A) according to the manufacturer’s directions. The reverse transcription and PCR of RNA were carried out sequentially in the same reaction plate using Verso 1-step RT-qPCR SYBR green kit (Thermo Scientific, AB-4104/A). RNA was added to a final concentration of 4 ng/μL. Intron-spanning primers (Table 1) were ordered from Bioneer Inc. Reactions were carried out in a Step One Plus Real-Time PCR System and cycle threshold (C_T_) analysis was completed with the Step One software using the ΔC_T_ method (version 2.2.2, Applied Biosystems). Amplification conditions consisted of 15 minutes of cDNA synthesis at 50°C followed by Thermo-Start activation at 95°C for 15 minutes. PCR was carried out in 40 cycles of denaturation for 15 seconds at 95°C, 30 seconds of annealing at 55°C, and 72°C extension for 30 seconds per cycle. The mRNA concentrations were calculated using C_T_ levels of target genes normalized to that of β-actin. Data was analyzed using ANOVA between groups where * = p < 0.05, ** = p < 0.005 and *** = p < 0.0005.

**Table 1:** List of primers used for real time RT-PCR.

Gene	Primer sequences	Size (bp)	Genbank Accession
GAPDH	Forward: 5’-AAGGTCHHTGTGAACG Reverse: 3’-GCTCCTGGAAGATGGTG	226	NM_008084.2
*KCNMB4*	Forward: 5’-GTGAACAACTCCGAGTCCAA Reverse: 3’-AGGAGAGCAATCTCGTCGT	248	NM_021452.1
Kcnma1	Forward: 5’-AATGTCTACAGTGGGTTATGG Reverse: 3’-AGTCCTTCAGGAAGTTAGAGA	236	NM_001253358.1
STREX	Forward: 5’-GCCAAAGAAGTTAAAAGGGC Reverse: 3’-ATCAAAACAACATGCTCGTC	141	NM_001253373.1

### Isolation of brain slices for electrophysiology

Brain slices were prepared from 23 to 28 day old mice. C57BL/6J strain and *KCNMB4* knockout mice inbred six generations into the C57BL/6J background were used [11]. Mice were deeply anesthetized with inhaled isoflurane (Baxter Healthcare Corporation) before decapitation and whole brain dissection. The brain was quickly placed into ice-cold cutting solution containing (in mM) 2 KCl, 2 MgSO_4_, 1.25 NaH_2_PO_4_, 1 CaCl_2_, 1 MgCl_2_, 26 NaHCO_3_, 206 Sucrose, 10 Dextrose, and 0.4 Vitamin C. The brain was mounted on a cutting platform where coronal brain sections were cut in a Letica vibratome to a thickness of 300 μm. The brain slices were then allowed to recover in extracellular solution at 30°C for one hour, and then maintained at room temperature until time for recording. The extracellular solution consisted of (in mM) 124 NaCl, 2 KCl, 2MgSO_4_, 1.25 NaH_2_PO_4_, 2 CaCl_2_, 26 NaHCO_3_, 10 Dextrose, and 0.4 Vitamin C. All solutions were continuously bubbled with a mixture of 95%/5% oxygen/carbon dioxide.

### Outside-out patch clamp recordings

The recordings were conducted at 32°C with an internal solution consisting of (in mM) 20 HEPES, 140 KMeSO_3_, and 2 KCl, pH 7.2. 5 mM nitrilo-triacetic acid and calcium was added to obtain a buffered 60 μM free Ca^2+^ internal solution. The calcium concentration was confirmed with a calcium-sensitive electrode. Cells were visualized with a 60 X NIR APO water-immersion objective (using a Nikon FN1 upright microscope and infrared light source) and only cells from the third to the fifth visible layer of the dentate gyrus were recorded in an outside-out configuration. Iberiotoxin (IBTX) (TOCRIS Bioscience) and paxilline (TOCRIS Bioscience) were diluted, to 100 nM and 5 μM respectively, in extracellular solution and exposed to membrane patches via a sewer pipe at a rate of 0.5 mL / minute. BK channels were presumptively identified due to their high conductance (~236 pS), reversal potential of ~ 0 mV (using symmetrical potassium), and voltage-dependent open probability. To detect β4 subunit association, IBTX sensitivity was assayed by external perfusion of IBTX for five minutes. To confirm that IBTX-resistant channels were indeed BK, they were subsequently blocked with paxilline (5 μM). Data was collected using a HEKA EPC10 amplifier and Patchmaster software (HEKA Instruments). Voltage was held at -60 mV and 20 mV steps of 5-second durations were collected. Channel open probability and dwell times were analyzed using TAC and TAC FIT software (Bruxton). The numbers of channels in the patch was revealed by using a +120 mV voltage command to evoke near-maximal opening of channels (in 60 μM free Ca^2+^ internal solution). Single channel data was idealized as outlined in [19]. Data is represented as averages ± S.E.M. and ANOVA was used to separate differences between groups.

### Whole-cell current-clamp recordings

Intracellular solutions consisted of (in mM) 120 K-gluconate, 10 HEPES, 0.1 EGTA, 20 KCl, 2 MgCl_2_, 2 Na_2_ATP, 0.25 Na_2_GTP, pH was adjusted to 7.4 with 1 M KOH. BK channels were blocked with a 10-minute perfusion of Paxilline (added to extracellular solution at a final concentration of 5 μM). Only cells visualized in the third to fifth layer of the slice were recorded. Cells that had a resting membrane potential more negative than -72 mV, and input resistances of 200–300 MΩ were analyzed. Cells were held with a holding current to maintain a resting voltage of -80 mV. Hyperpolarizing input resistance (HIR) was measured using a -20 pA step below the holding current and depolarizing input resistance (DIR) was calculated using an 40 pA step above the holding current (Table 2). Voltage traces were collected using a HEKA EPC10 amplifier and Patchmaster software (HEKA Instruments). During whole-cell current clamp, cells receive both the -80 mV holding current and additional stepwise increases of 40 pA steps (lasting 1000 ms) with 10 s pauses between seven steps. The individual properties of action potential waveforms were analyzed using AxoGraph software (Kagi, Berkeley, CA). Action potentials were detected with a derivative threshold of 20 mV/ms. Rise times were calculated from 10 to 90% of the peak, and action potential width was measured at 10% of action potential peak. Fast-afterhyperpolarization amplitude was measured as the maximum negative peak following an action potential (always occurring less than 2ms after spike) minus the threshold of action potential initiation. Parameters of action potential properties are reported as the average values obtained from three sequential voltage recordings at the 200 pA (above holding potential) current injection. Data analysis of the averaged values from single cells, before and after 10 minutes of paxilline perfusion, are treated as paired data with statistical significance being reported using a Student’s t-test. As a control to ensure that paxilline evoked changes in action potentials properties were not due to dialysis of cells, we conducted time control experiments. Time dependent (>10 minutes) changes in fAHP (0.5 mV ± 0.17 mV), threshold (-1.8 ± 1 mV), and instantaneous frequency (+8.6 ± 12 Hz) were significantly smaller (P <0.05, N = 6) than paxilline effects reported. Comparison of BK specific (paxilline) changes in control and seizure (pilocarpine) values was done with a single-sample Student’s t-test. Comparisons of average changes between control and seizure groups were completed with an ANOVA. P-values are represented as * = p < 0.05, ** = p < 0.005, and *** = p < 0.0005.

**Table 2:** Averages of subthreshold voltage trace data.

	N	RMP (mV) ± s.e.m	HIR (MΩ) ± s.e.m	DIR (MΩ) ± s.e.m
Control	9	-89 ± 2	637 ± 92	747 ± 73
Control + Paxilline	9	-89 ± 2	623 ± 90	657 ± 50
Seizure	8	-85 ± 4	435 ± 56	588 ± 57
Seizure + Paxilline	8	-85 ± 4	470 ± 40	571 ± 47

The average ± S.E.M. of resting membrane potential (RMP) hyperpolarizing input resistance (HIR), and depolarizing input resistance (DIR). Analysis using paired Student’s t-test shows significance between Control and Control + Paxilline DIR (p = 0.02).

### Kainic acid challenge and video-electrocorticography (ECoG) recordings

*KCNMB4* heterozygous mice for kainic acid sensitivity and contextual fear conditioning were littermates (different mice were used for the two assays) from a C57BL/6J and *KCNMB4* heterozygous cross. Kainic acid sensitivity and ECoG recordings were conducted according to [20]. Electrical activity was recorded using four screw-type subdural electrodes via prefabricated headmounts (Pinnacle Technologies). During recordings, a 25X preamplifier was connected to the headmount, and this was connected via wires through a commutator to a Stellate Harmonie Amplifier EEG system that synchronized behavior video recordings with the EEG tracings. The left-frontal screw was used as a common reference, left temporal (top channel in tracings), right frontal (middle channel), and right temporal screws (bottom channel) were recorded. Mice were allowed to recover at least one week before the ECoG recordings. The investigator was blind to the genotype of the mice when seizure frequency and duration were analyzed. Comparison of time to seizure, and total seizure time was conducted with an unpaired student t-test.

## Results

### Seizure activity reduces β4 subunit expression

Here we investigated acute (2 day) post-seizure changes in BK channel pore-forming α subunit and its neuronal accessory β4 subunit. Given a predominant expression of these two subunits in the hippocampus, we focused our study on this region. Mice were treated with pilocarpine to induce stage four seizures (see Methods) [17]. RT-qPCR analysis of dissected hippocampi showed a 41% reduction of β4 mRNA in pilocarpine-treated animals (Fig 1A). In order to demonstrate that β4 reduction resulted from seizure activity rather than pilocarpine alone, we repeated this experiment with kainic acid treated mice and observed very similar results (Fig 1A). Similar effects from two different chemoconvulsants indicate that increases in neuronal excitability, rather than the pilocarpine or kainic acid per se, underlie decreases in *KCNMB4* mRNA expression.

We also assessed BK α subunit mRNA levels and determined if changes in alternative splicing of the stress-regulated exon (so called “STREX” exon [21]) also occurs following a seizure. Previous studies have demonstrated that STREX expression is increased 10 days following seizures [22], but this alternative splicing event has not been documented at the 48-hour time point. RT-qPCR of STREX indicated that although there is a 30–50% decrease in STREX expression (Fig 1C), this is accounted for by a similar decrease in total BK mRNA expression (Fig 1B). Indeed, normalizing STREX to total BK mRNA yielded no changes in relative STREX expression (Fig 1D). Although BK α mRNA levels are reduced (Fig 1B), immunostaining did not detect a change in BK channel protein in the dentate gyrus as measured by immunohistochemistry (Fig 1E, summarized in Fig 1F). In summary, BK channels two days following a seizure demonstrated a reduction of both BK α and β4 subunit mRNA, although protein levels of α were not altered.

**Fig 2:**
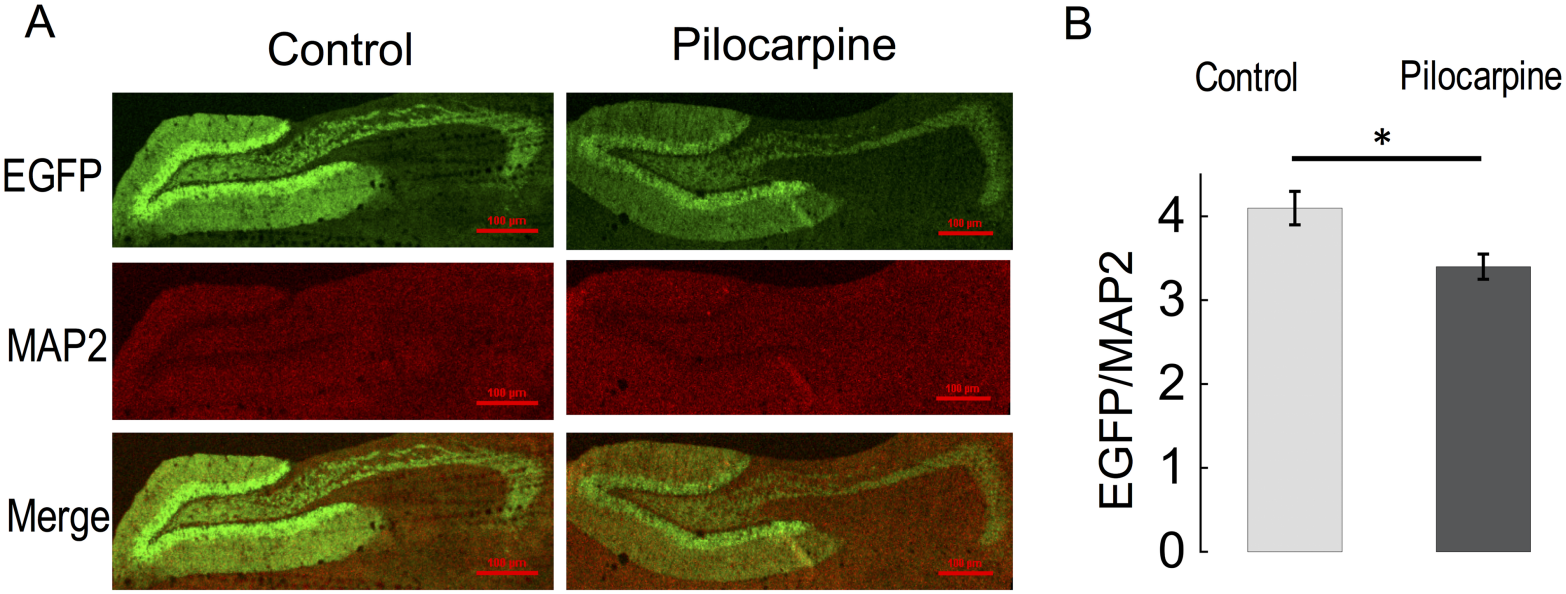
Pilocarpine-induced seizures reduce *KCNMB4*-EGFP reporter expression. (A) Anti-EGFP (top panels) and anti-MAP2 (middle panels) immunofluorescence staining from heterozygous mice containing the EGFP cDNA that replaces the first coding exon of *KCNMB4* gene [10]. The pictures are oriented from left, medial (the dentate gyrus) to right, temporal (hilar mossy fibers extending to CA3 region). Bottom panels are merged of above images. (B) Average fluorescence intensity of EGFP normalized to MAP2 measured at the granule cell area. Control is 4.1 ± 0.20, N = 7, pilocarpine 3.4 ± 0.18, N = 6, P = 0.034.

While we were unable to identify β4-specific antibodies useful for immunostaining, we nevertheless could corroborate changes in *KCNMB4* mRNA described above using an EGFP reporter that is gene-targeted to the *KCNMB4* locus [10]. This allowed us to determine if chemoconvulsant reduction of *KCNMB4* gene expression specifically occurs in dentate gyrus granule neurons (neurons that we analyzed using electrophysiology in subsequent experiments). Heterozygous EGFP- *KCNMB4* knockin mice were treated with pilocarpine as described above, and brain sections were co-stained with anti-EGFP antibodies to assay *KCNMB4* gene expression and anti-MAP2 to normalize for tissue density (Fig 2A, top panels). MAP2 signals in control and pilocarpine treated mice were not significantly different (Fig 2A. control 1227 ± 268 N = 7, pilocarpine 1363 ± 245 N = 6, P = 0.72, middle panels). We found a significant decrease in EGFP fluorescence in pilocarpine-treated mice (summarized in Fig 2B, control 4.1 ± 0.20, N = 7, pilocarpine 3.4 ± 0.18, N = 6, P = 0.034), albeit not to the extent of mRNA reductions assayed by PCR, perhaps due to a slower turnover of conventional EGFP protein [23]. As a positive control, we also evaluated BAC transgenic mice that contain EGFP targeted to the KCNQ2 (Kv7.2) locus. Previous studies indicate an increase in hippocampus KCNQ2 mRNA expression following seizures (Zhang and Shapiro, 2012). We indeed found a pilocarpine-induced increase in CA3 expression of KCNQ2 (but not dentate gyrus expression, panels A and B in S1 Fig). The EGFP reporter detects changes in expression (2-fold) that is less than that measured by RT-PCR (28 fold) (Zhang and Shapiro, 2012).

**Fig 3:**
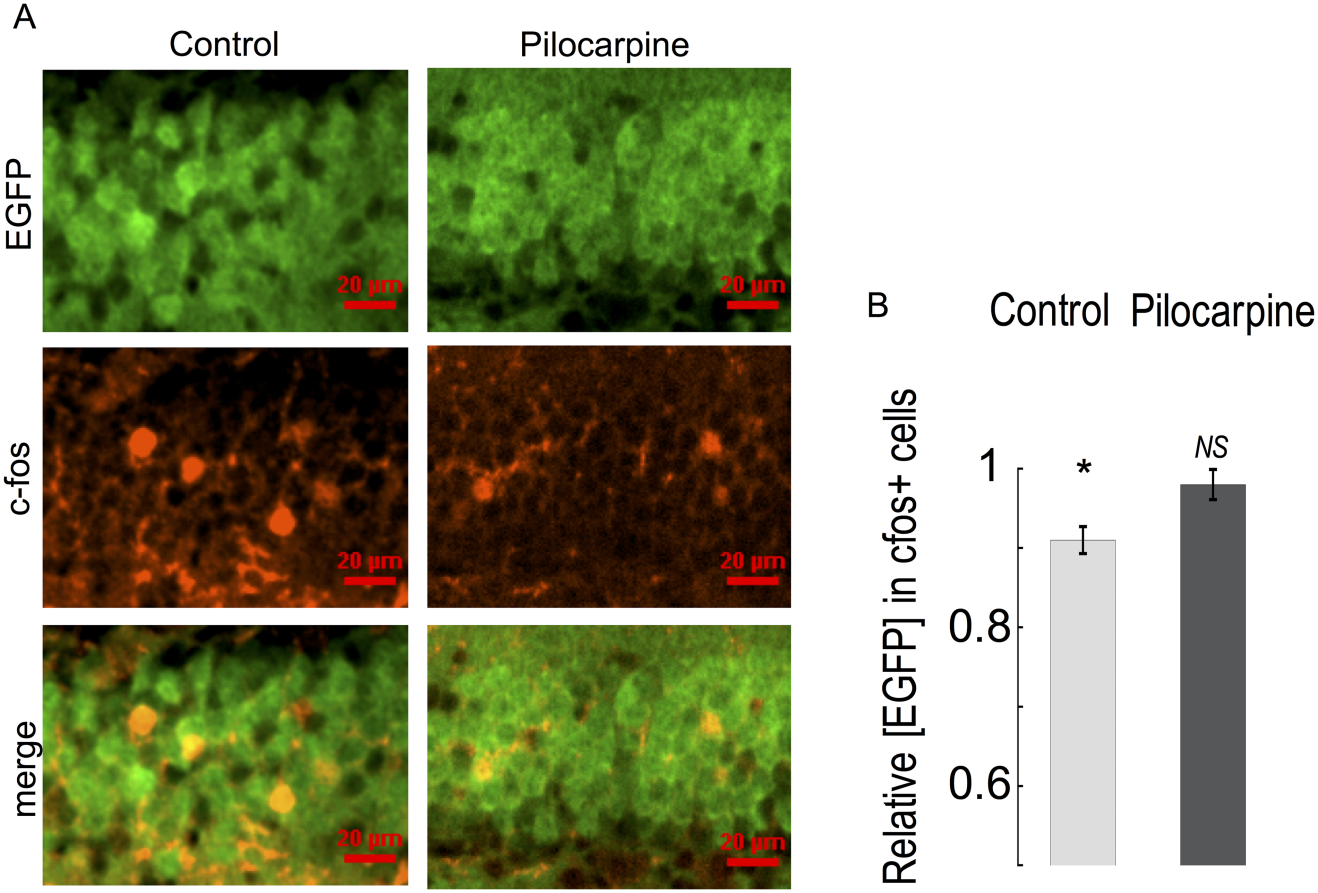
cfos immunostaining marks neurons with reduced gene expression of *KCNMB4*. (A) Anti-EGFP (top panels) and anti-c-Fos (middle panels) immunofluorescence staining from heterozygous mice containing EGFP knockin into the *KCNMB4* locus [10]. Left panels are control mice, right panels are pilocarpine treated mice. The pictures are focused on the granule cell layer of the dentate gyrus. Bottom panels are merged images from panels above. (B) Average anti-EGFP immunofluorescence in c-Fos positive cells normalized to EGFP immunofluorescence in c-Fos negative, neighboring cells. Control cells show a reduced EGFP immunofluorescence in c-Fos positive cells (bar value) normalized to c-Fos negative cells in the section (0.91 ± 0.017, N = 12, P < 0.001). Pilocarpine-treated mice show no reduction in EGFP immunofluorescence in c-Fos positive cells relative to c-Fos negative cells. (0.98 ± 0.019, N = 15, P = 0.38).

The above results indicate that changes in excitability may cause changes in *KCNMB4* gene expression. We therefore investigated if c-Fos immunoreactivity (that reports neurons with recent activity [24]) marked neurons that have a reduction of *KCNMB4* expression in the absence of chemoconvulsant treatment. Co-immunostaining of c-Fos with *KCNMB4* EGFP protein is shown in Fig 3. A comparison of *KCNMB4* EGFP in c-Fos positive cells relative to neighboring c-Fos negative neurons in the section indicated reduced *KCNMB4* expression to an average 91.0% of c-Fos negative cells (Fig 3A, summarized Fig 3B, 0.91 ± 0.017, N = 12, P < 0.001). However, pilocarpine-induced downregulation of *KCNMB4* expression (Fig 2) occluded any further reduction of *KCNMB4* EGFP in c-Fos positive cells (Fig 3A, summarized Fig 3B, 0.98 ± 0.019, N = 15, P = 0.38).

### Seizure activity reduces iberiotoxin-resistant BK channels

**Fig 4:**
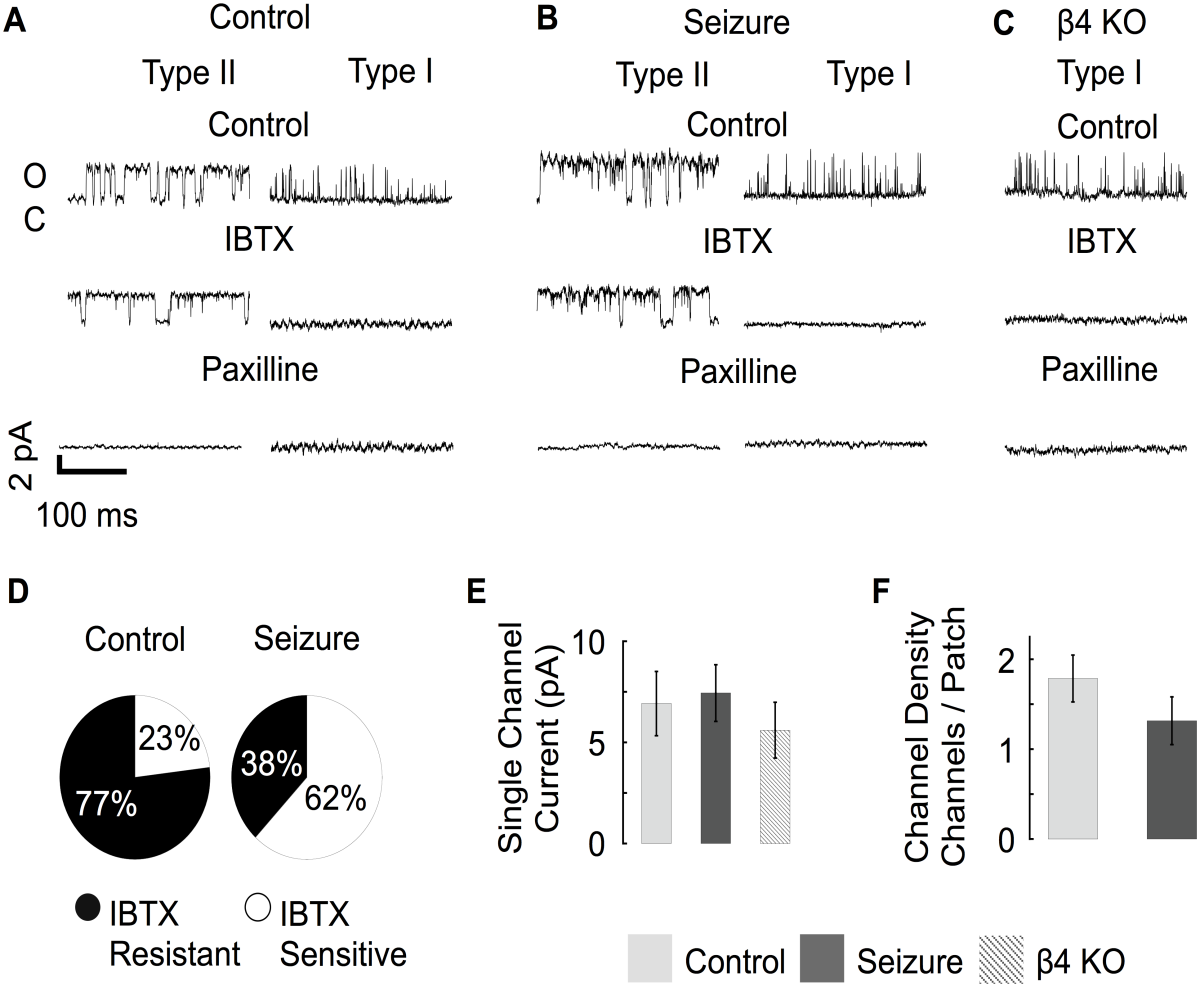
Control and seizure animals express different ratios of type II and type I BK channels. (A-B) Type II channels have longer open dwell times and were resistant to IBTX. Type I channels had observably shorter open dwell times and were consistently blocked by IBTX. All channels were blocked by paxilline. (C) β4 KO mice only express Type I channels that are sensitive to IBTX and paxilline. (D) Type I BK channels were more predominant from seizure experienced mice (Control n = 15, Seizure n = 19). (E) Single channel current amplitude was similar in control, pilocarpine treated, and β4 KO mice. Single channel current was an average of 7 ± 1.5 pA at a 0 mV holding potential. (Control n = 8, Seizure n = 7, β4 KO n = 5) (F) The average number of BK channels per membrane patch was similar in control and seizure mice. (Control n = 15, Seizure n = 19). Panels A-E data was acquired at 0 mV potential with 60 μM buffered calcium internal solution.

Given we observed downregulation of *KCNMB4* mRNA in dentate gyrus granule neurons after seizures, we conducted outside/out patch clamp recording to determine if BK channel properties were altered. In addition, single channel recording provides a clear indicator of β4 subunit association as evidenced by a resistance to iberiotoxin block [25]. Following excision, all patches were analyzed for channel conductance, gating kinetics and open probability. Conductances believed to originate from BK channels were verified by confirming their sensitivity to iberiotoxin (to block BK channels lacking β4 subunit [25]), and paxilline (antagonist of all BK channels [26]). Furthermore, the outward currents were relatively large showing an average slope conductance of ~236 pS (R^2^ = 0.99 from -40 to 40 mV) [27]. Channel properties measured from dentate neurons from control and seizure-experienced animals had the pharmacological profile of a mixture of both iberiotoxin-sensitive, type I and iberiotoxin-resistant, type II BK channels (Fig 4A and 4B). Qualitatively we noticed that BK channels that were resistant to IBTX (type II) remained open for a longer duration when compared to IBTX sensitive channels (Fig 4A and 4B). In neurons from *KCNMB4* knockout mice, BK channels were blocked by IBTX (Fig 2C). Consistent with previous studies [10], most channels were iberiotoxin resistant in control granule neurons (77%, Fig 4D). In contrast, most channels from seizure treated mice were iberiotoxin-sensitive (62%, Fig 4D). There was no quantifiable difference in the single channel conductance of BK channels from control, seizure experienced, or *KCNMB4* KO animals (Fig 4E). Consistent with immunohistochemical studies (Fig 1E), we were also unable to resolve a difference in the density of BK channels as measured by the average number of channels per patch (Fig 4F).

**Fig 5:**
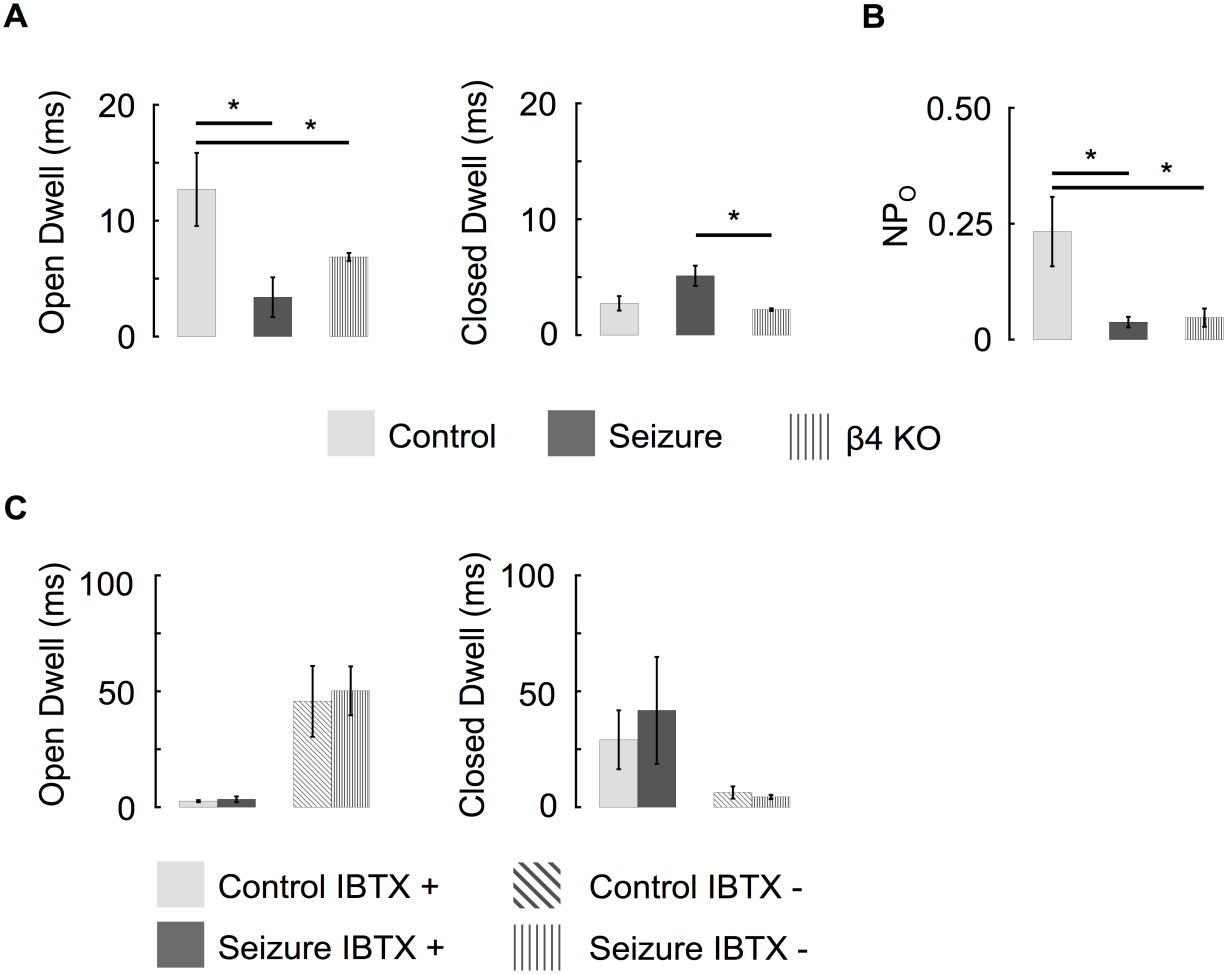
Seizure activity switches predominant BK channel gating properties from type II to type I. (A) The average open dwell time of BK channels recorded from pilocarpine treated mice was more similar to β4 KO channels than they were to control recordings. (B) The seizure driven loss of β4 reduces the NP_O_ of channels at a -40 mV holding potential. (Control n = 8, Seizure n = 7, β4 KO n = 5) (C) Independent of seizure history, the dwell times of IBTX sensitive (and IBTX resistant) BK channels were similar. (Control IBTX sensitive n = 4, Control IBTX resistant n = 6, Seizure IBTX sensitive n = 5, Seizure IBTX resistant n = 4). Recordings were made with 60 μM buffered calcium internal solution.

Analysis of channel gating showed that BK channel open dwell-times following seizure were more similar to *KCNMB4* KO recordings than control animals (Fig 5A). We also noted that, similar to *KCNMB4* KO, the open probability of single BK channels was lowered following pilocarpine (Fig 5B). Thus, reduced iberiotoxin resistance, reduced P_o_, and faster gating kinetics are all consistent with a reduction of β4-containing BK channels following seizures. Separation of BK channels based on their IBTX sensitivity revealed similar gating properties that were independent of seizure history (Fig 5C). We conclude that the association between the BK channels α and β4 subunit is decreased following seizure activity and results in the predominant expression of type I channels in granule neurons.

### Seizures do not affect early spike frequency

**Fig 6:**
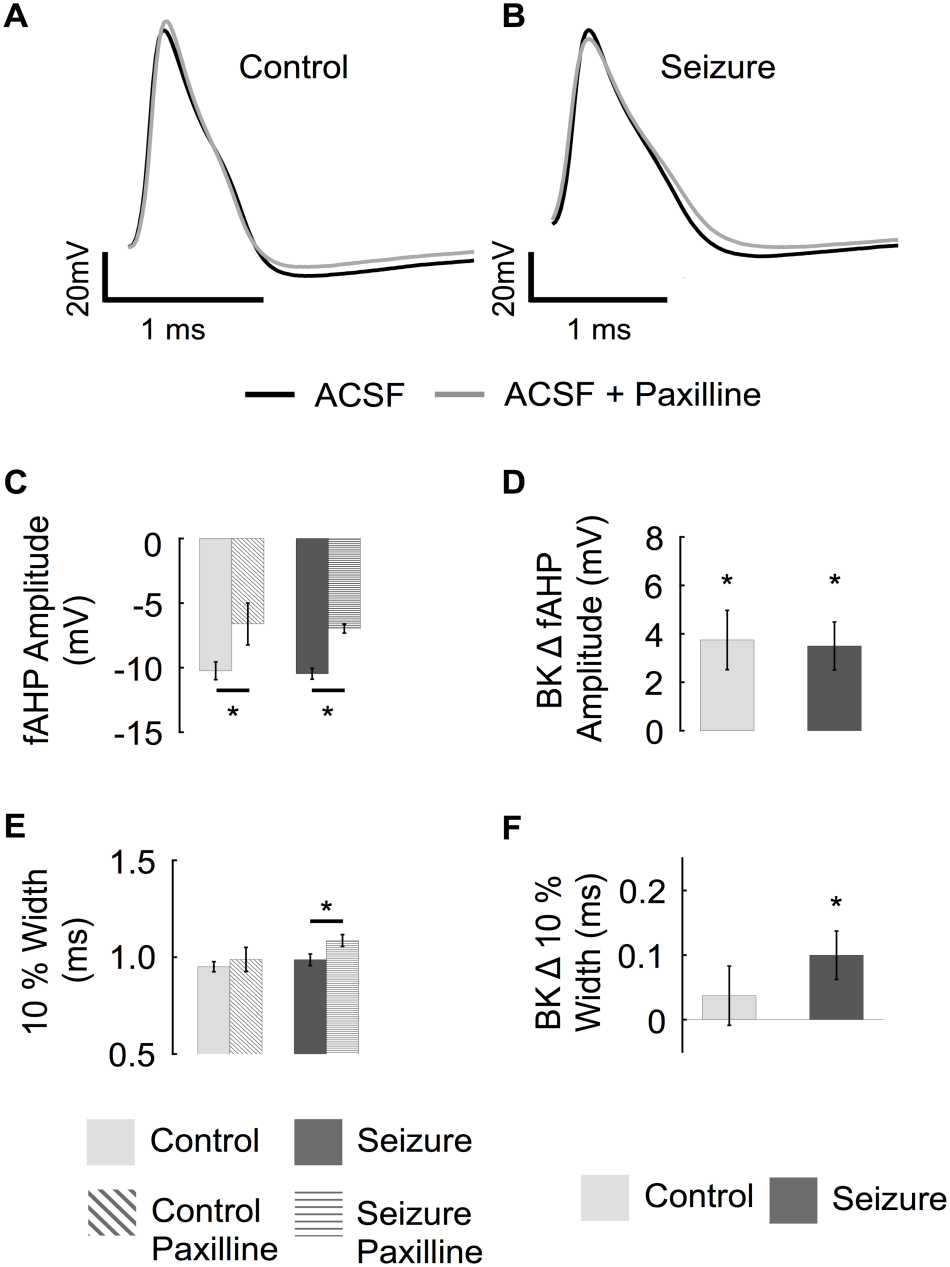
BK channels shape the action potential afterhyperpolarization. (Top) Representative traces of 1^st^ action potential comparing BK channel block (paxilline, grey trace) in control (A) and pilocarpine treated (B) granule cells. (C) Average fAHP amplitude before and after paxilline from control and seizure experienced animals. (D) The relative effect of paxilline on fAHP amplitude. (E) Average 10% width of first action potential before and after paxilline from control and seizure experienced animals. (F) The relative effect of paxilline on the 10% width of the first action potential. (Control n = 9, seizure n = 8).

In order to understand the physiological consequences for a reduction in β4 and type II BK channels, we conducted current clamp studies to analyze changes in intrinsic excitability. Past studies have demonstrated several adaptive and maladaptive changes in ionic currents that follow seizures [1–5]. Therefore we discerned changes specific to BK channels by conducting paired experiments comparing before and after treatment with BK channel blocker paxilline in each neuron. In addition, we recorded neuronal activity at 32°C rather than room temperature in order to emulate physiological conditions. Under these conditions, we find that wild type neurons expressing BK/β4 channels do not have an apparent affect on the repolarization phase but do increase the fAHP amplitude (Fig 6A). Following seizure activity, the BK channel contribution to fAHP remained, as evidenced by a similar paxilline-sensitive fAHP amplitude (Fig 6C and 6D). However, the seizure mice showed an increased paxilline-sensitive AP width, indicative of a greater recruitment of BK channels during the repolarization phase (Fig 6E and 6F).

**Fig 7:**
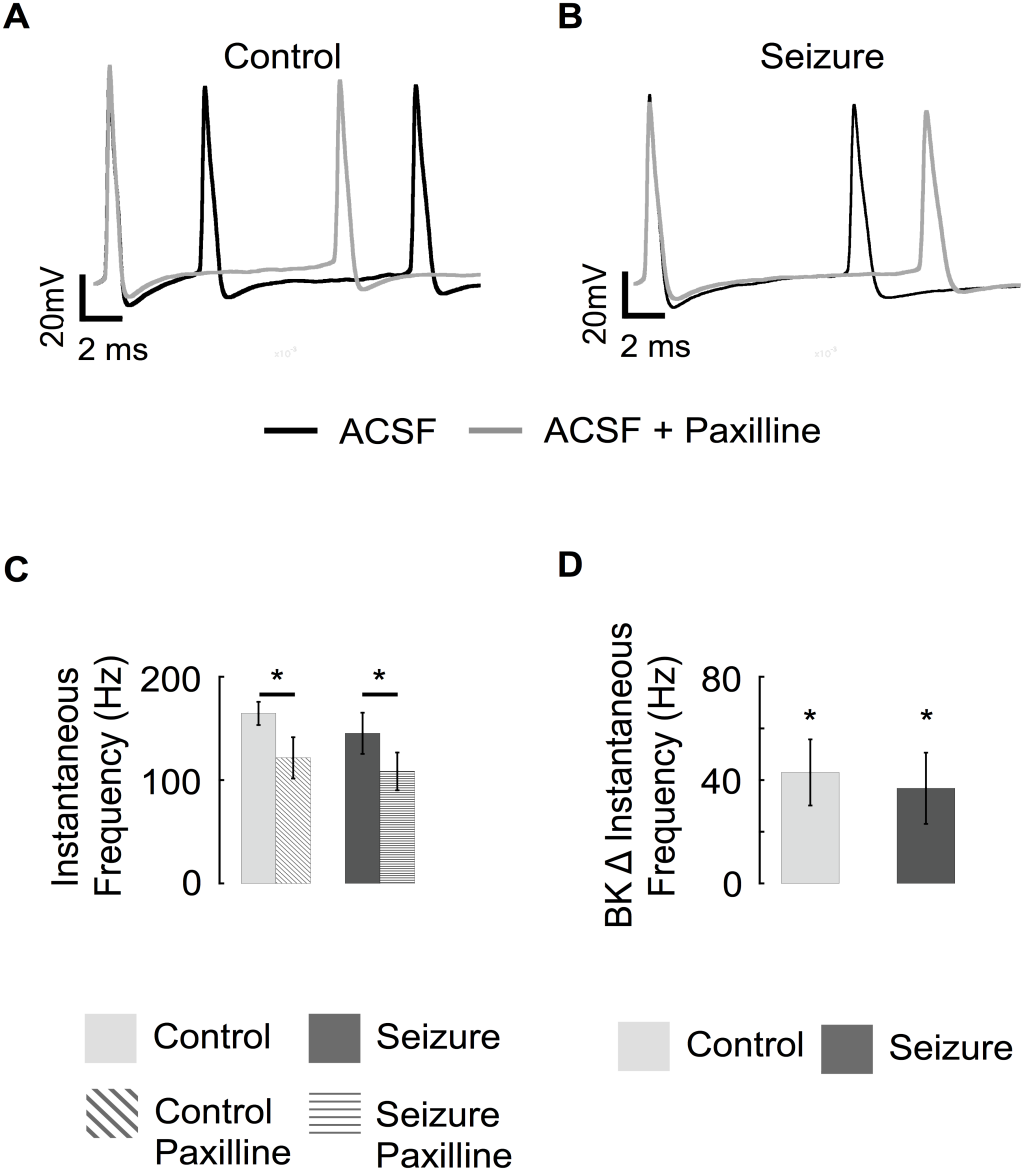
Seizures do not change BK channel contribution to early spike frequency. (Top) Representative traces of first 20 ms of voltage response to a 200 pA constant current injection. The voltages traces are aligned at the first spike (pre-spike tracings are not shown) to display effects on instantaneous frequency. Before (black trace) and after BK channel block (paxilline, grey traces) in control (A) and pilocarpine treated (B) granule cells. (C) Average instantaneous firing frequency of 1^st^ action potential before and after paxilline from control and seizure experienced animals. (D) Average change in instantaneous firing frequency resulting from blocking BK channels in control and pilocarpine treated mice. (Control n = 9, seizure n = 8).

The consequence of β4 downregulation on spike frequency before and after seizures was investigated (Fig 7). Similar to CA1 neurons [28], granule neuron BK channels increase early spike frequency, as evidenced by reduced instantaneous frequency (longer 1^st^ and 2^nd^ spike intervals) following BK channel block with paxilline (Fig 7A, summarized in Fig 7C). Following seizures, the effect was relatively similar (Fig 7B), with a decrease in instantaneous frequency by 40 Hz (Fig 7D) following paxilline block of BK channels. In conclusion, β4 downregulation following seizures shows an effect on AP repolarization, but no significant effect on AP frequency.

### BK channels increase the action potential threshold

**Fig 8:**
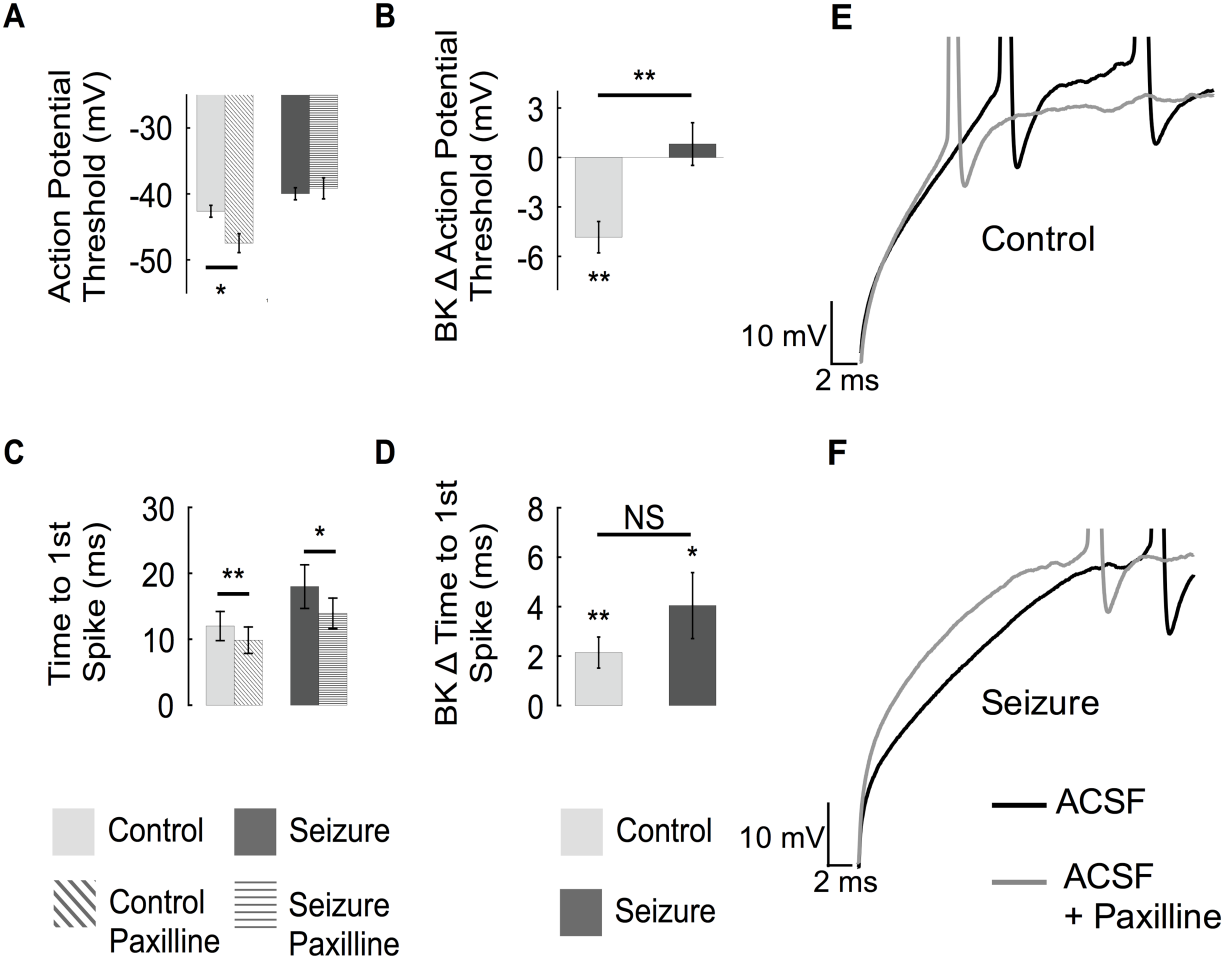
BK channels affect the action potential threshold and delay to first spike. (A) The first action potential threshold voltage is hyperpolarized following paxilline in control mice (A, left), while there is no effect of blocking BK channels in seizure-experienced neurons (A right). (B) Average of absolute change in threshold potential of first spike in response to paxilline block in control and seizure treated mice. (C) The average time to spike is decreased by paxilline in control and seizure treated animals. (D) The average absolute change in time to spike resulting from BK channel block from control and seizure treated animals. (E, F) Representative voltage traces showing the change in threshold and time to first action potential for control recordings (E) and recordings after seizure (F). (Control n = 9, seizure n = 8).

Analysis of voltage traces during current injection (200 pA from holding at -80 mV) showed an average AP threshold of -42 mV ± 1 for the first spike. In paired experiments, blocking BK channels shifted the AP threshold to an average -47 mV ± 1 (Fig 8A and 8B), suggesting that BK channels oppose the rise to threshold. This effect was also observed using near-threshold current injections and also in the presence of glutamate and GABA_A_ receptor antagonists, kynurenic acid and picrotoxin, respectively (control -45.6 ± 1.3 versus -49.4 ± 2.2 with paxilline, P = 0.03). In contrast, two days following seizures, BK channels did not affect the spike threshold voltage (Fig 8A and 8B). This loss of BK channel threshold function may be explained by downregulation of the β4 subunit, as the interaction of β4 with the BK α subunit has shown to increase the P_o_ of BK channels (Fig 3B). Interestingly, following seizures a compensatory depolarization of AP threshold was observed that is independent of BK channels (Fig 8A, right panel). An additional subthreshold effect of BK channels observed was a paxilline-sensitive delay of time to first spike (Fig 8C and 8D). In contrast to effects on threshold, this effect was not lost following seizures. In summation, reduction in *KCNMB4* expression following seizure activity (Fig 1) correlates with a reduction of type II BK channels, typically exhibiting a high P_o_ (Fig 3), and a loss of BK channel effect on AP threshold (Fig 8). Meanwhile, despite seizures and β4 downregulation, BK channels effects on time to first spike, fAHP and interspike intervals are retained.

**Fig 9:**
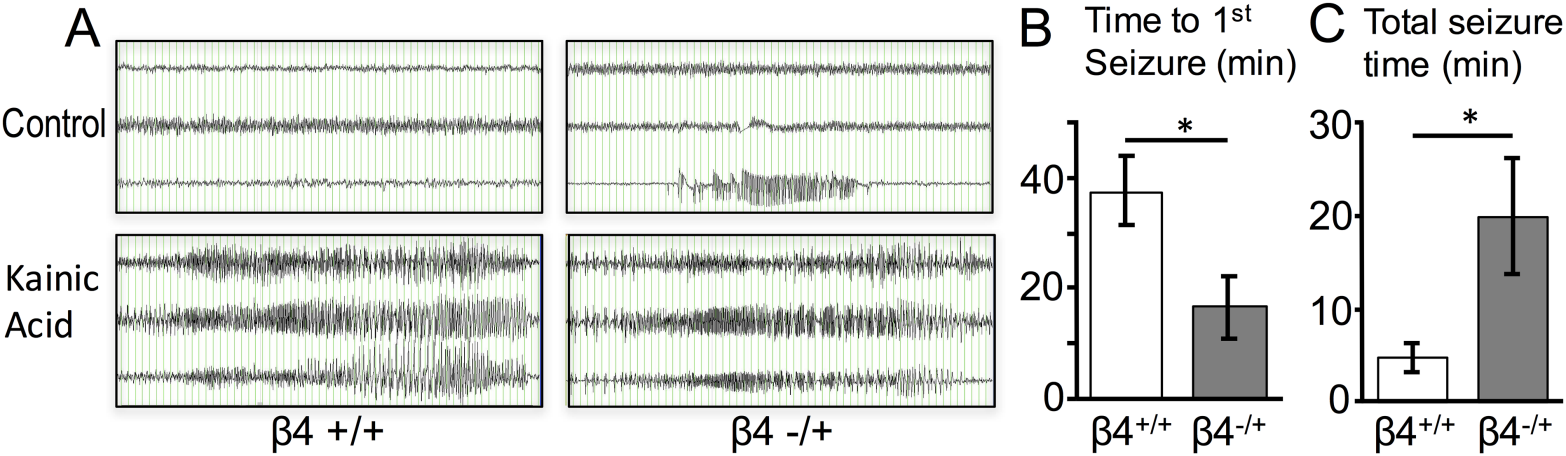
Reduced β4 expression has consequences on seizure threshold. (A) EEG recordings of mice monitored for 24 hours for spontaneous EEG activity (top panels) and in response to cumulative increased doses of kainic acid (KA, 10 mg/Kg) at 30 minute intervals (bottom panels). Wild type β4^+/+^ do not have spontaneous seizures. One heterozygous β4^-/+^ had a spontaneous focal seizure (above) and had generalized seizures (bottom) within 10 minutes of a single dose (10 mg/Kg) of KA. β4^+/+^ control mice required a second dose of kainic acid (20 mg/Kg total) to demonstrate seizures (bottom left). Time scale is 1 second per division. The channels recorded were from left temporal (top channel), right frontal (middle channel), and right temporal (bottom channel). B. Average time to 1st seizure and C. total seizure time after KA in β4^+/+^ and β4^+/-^ mice (N = 7 each β4^+/+^ and β4^+/-^).

We undertook a genetic approach to determine if β4 reduction has physiologically consequences by using heterozygous mice to reduce gene expression. Previous studies indicate β4^-/+^ mice have an approximate 50% reduction in mRNA compared to wild type [10], similar to the reduction seen after seizures. After 24 hours of video-EEG recording, we observed that one of seven β4^-/+^ had spontaneous focal seizures (Fig 9A top right panel). We challenged mice with increasing doses of kainic acid and observed a dramatic increase in kainic acid sensitivity with reduced β4 expression. Doses that did not cause electrographic seizures in wild type siblings (10 mg/Kg) caused severe seizures in β4^-/+^ mice within minutes of the first dose (Fig 9B) and also for longer durations (Fig 9C).

## Discussion

### *KCNMB4* transcription is regulated by neuronal activity

Early single-channel studies from synaptosomal preparations identified two major types of neuronal BK channels: fast-gated, iberiotoxin sensitive type I channels and slow-gated, iberiotoxin resistant type II channels [29]. Heterologous expression and knockout studies of *KCNMB4* indicated that many type II BK channel properties, including pharmacology and gating kinetics, were conferred by BK channel association with the β4 accessory subunit [10, 25, 30–32]. Here we show that the abundance of β4, and therefore type II BK channel subtypes, is reduced, presumably through increased excitability, 48 hours following chemoconvulsant treatment with two different pro-excitatory compounds that are commonly used to initiate development of hippocampus-dependent, temporal lobe epilepsy [13–15]. This resulted in a switch from type II to predominantly type I, fast-gated BK channels two days following seizures. Given that β4 protects against epilepsy [10], downregulation of β4 suggests a maladaptive change in BK channels that may further promote seizures. Consistent with this, *KCNMB4* heterozygous mice, which have reduced β4 mRNA expression, have an increased sensitivity to kainic acid induced seizures (Fig 9).

### BK channels facilitate firing frequency in granule cell neurons

Previous studies using recording conditions at room temperature indicated that BK channels in DG neurons did not shape action potentials or affect spike frequency [10, 11, 33]. However, temperature can affect channel’s gating kinetics. In this study, recordings were gathered at the more physiological temperature of 32°C. This revealed that BK channels in DG neurons did affect the fAHP amplitude but not the action potential repolarization phase. As previously shown in CA1 neurons [28], we found that BK channels decrease the early interspike intervals to increase excitability in granule cells. However, we were surprised to find that type II BK channels are also active preceding the spike to depolarize action potential thresholds. Thus, we find that BK channels in DG neurons have dual effects on excitability. Although they would be expected to oppose AP firing during an EPSP, once an action potential occurs BK effect on the fAHP promotes additional subsequent APs.

Following seizures, downregulation of β4 reduces BK channel open probability, which may underlie the loss of BK subthreshold inhibitory effects while maintaining BK channel pro-excitatory effects on spike frequency. Interestingly, Mehrenfard et al. have also studied BK channel effects after pilocarpine-induced seizures (24 hours) in rat DG neurons, and observed a gain-of-function of BK channels apparent as an increased paxilline-sensitive sharpening of action potentials and increased fAHP amplitudes, consistent with a downregulation of the β4 subunit [33]. However, while we did not see a pilocarpine effect on BK facilitation of spike frequency, Mehrenfard et al. did [33]. The distinction between the two studies could be recording conditions such as temperature, or the use of internal solution with (this study) or without calcium buffering [33]. Nevertheless, both studies are consistent with effects explained by a downregulation of the β4 subunit.

### BK channels habituate the initial action potential discharge

Effects of BK channel blocker paxilline on action potential threshold suggest that type II BK channels are activated at subthreshold potentials in dentate gyrus granule neurons. Downregulation of subthreshold A-type potassium currents was also observed in DG neurons following hypoxia-induced seizures [1]. Subthreshold activation is a property not generally ascribed to neuronal BK channels. BK channels generally affect membrane voltage only during or immediately after action potentials [11, 34, 35]. However, superchiasmatic neurons show a depolarization of resting potential in BK knockout mice [36]. Further, BK channels are shown to be coupled to low-voltage, T-type calcium channel which mediate BK activation at negative voltages (-50 mV) in medial vestibular neurons in rats [37]. Recent studies revealed that β4 indeed confers a dramatically increased open probability to BK channels when physiological potassium solutions are utilized for recordings [38, 39] suggesting that α/β4 might be active preceding the spike. This was corroborated in this study of DG neurons that showed that iberiotoxin-resistant (type II) channels have a much higher open probability than iberiotoxin-sensitive (type I) channels. Two days following seizure-induced downregulation of β4, reduced numbers of type II and increased type I BK channels, there was a correlated loss of BK channel effect on threshold.

An alternative explanation to the changes in subthreshold BK channel effects may be related to their subcellular distribution. Given dense BK channel expression in the hippocampal mossy fibers [40], it is possible that BK/β4 may be enriched in the axon to oppose sodium channel activation preceding a spike. This is analogous to Kv7 channels that are enriched at the axon initial segment, and in a similar manner as type II BK channels, hyperpolarize action potential threshold voltages [41]. High resolution microscopy of somatic BK channels has found two distinct pools of BK channels: those that are scattered, and those that are clustered and frequently appose submembrane cisternae [42]. In control neurons that contain a greater abundance of type II BK channels, membrane patches were more often associated with a single BK channel which might reflect the scattered pool of BK channels. In seizure mice that contain greater type I BK channels, excised channels showed a greater number of empty patches although patches with 2 and 3 channels were often observed suggesting a more clustered organization. Whether the scattered pool/type II BK channels has a greater localization in the axon remains to be determined, although others have observed an iberiotoxin-resistant (type II) BK channel in recordings of mossy fiber terminals [43]. Thus, one could speculate that the effect of seizures is, in part, to move BK channels away from regions that affect spike initiation.

Granule cells undergo extensive ion channel reorganization during epileptogenesis, and BK channels are likely to be one of many other channels that are altered by seizure activity. Although we observed a seizure-dependent loss of BK channel effects on depolarizing input resistance, adaptive mechanisms following seizures appear to confer a BK channel independent decrease of input resistance. In part, this may be due to gain of inward rectifier K^+^ channel (Irk/Kir2) expression that is seen in epileptic neurons [3]. It may be concluded that the observed sub-threshold BK channel loss-of-function may be later resolved by the upregulation of resting K^+^ conductance in epileptic neurons.

### A possible role for the downregulation of *KCNMB4* in plasticity

The physiological function of ion channels is often highlighted in the study of the channel’s function in a pathological process. The initial shift from the predominant expression of type II to mostly type I BK channels may be an artifact of short-term plasticity that is activated by epileptogenic compounds. Increased spiking that results from seizure activity also activates learning dependent pathways [44]. This study also suggests a plausible role for BK channels in the activation of memory circuits. It shows that increased excitability leads to the genetic downregulation of type II BK channels. By decreasing the P_o_ of the channel, the loss of β4 association removes BK channel opposition to spike generation. The result of this situation would be an increase in the excitability of memory-associated circuitry within the hippocampus.

## Supporting information

S1 Fig (A) Anti-EGFP (top panels) and anti-MAP2 (middle panels) immunofluorescence staining from KCNQ2 Bac transgenics containing the EGFP reporter [10]. The pictures are oriented from left, temporal CA3 region, and right is the dentate gyrus. Bottom panels are merged of above images. (B) Average fluorescence intensity of EGFP normalized to MAP2 measured at the CA3 and granule cell area.
(TIFF)

## Abbreviations

AP: action potential
BK: large conductance voltage- and calcium-activated potassium channels
DG: dentate gyrus
**fAHP**: fast-afterhyperpolarization
