# Supplementary figures and images for: Downregulation of KCNMB4 expression and changes in BK channel subtype in hippocampal granule neurons following seizure activity
Source: PLoS One. 2017 Nov 16;12(11):e0188064. doi: 10.1371/journal.pone.0188064 (PMC5690595; doi:10.1371/journal.pone.0188064)

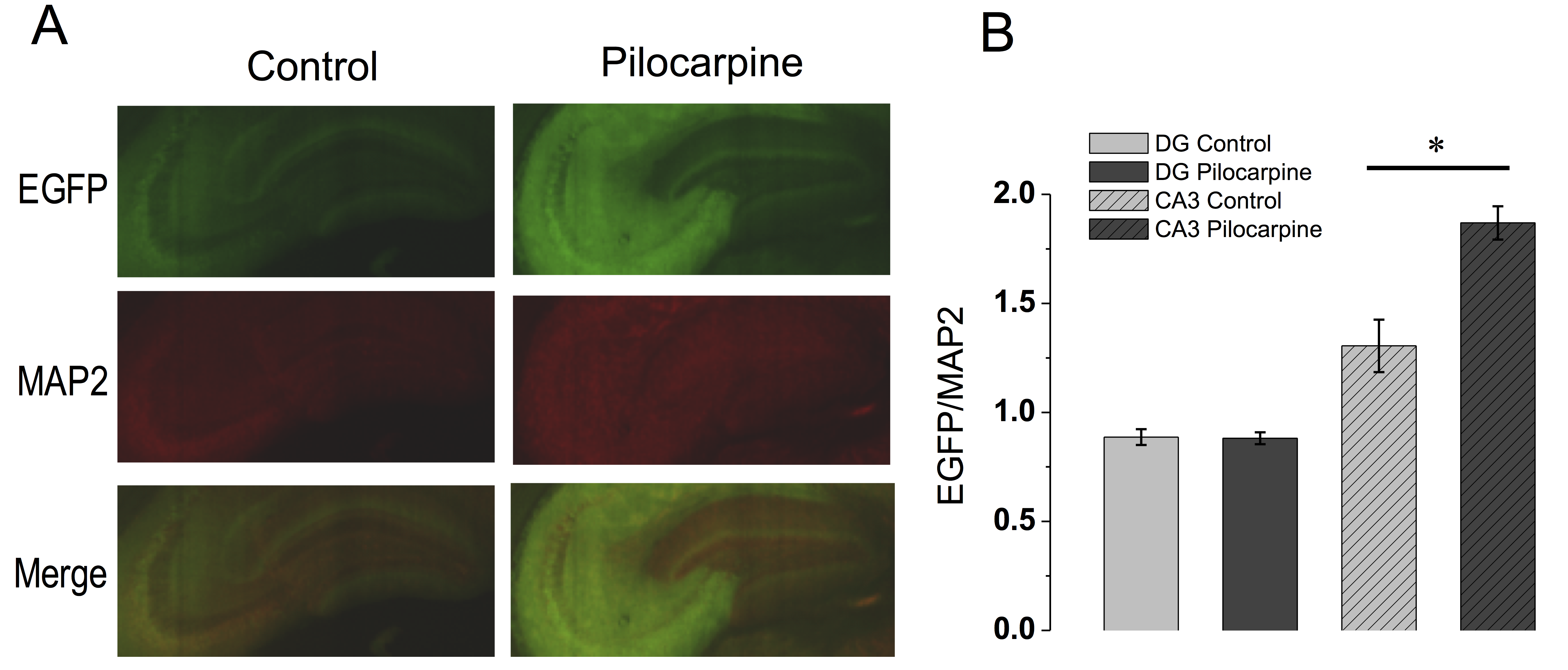

Supplement: S1 Fig — (A) Anti-EGFP (top panels) and anti-MAP2 (middle panels) immunofluorescence staining from KCNQ2 Bac transgenics containing the EGFP reporter [10]. The pictures are oriented from left, temporal CA3 region, and right is the dentate gyrus. Bottom panels are merged of above images. (B) Average fluorescence intensity of EGFP normalized to MAP2 measured at the CA3 and granule cell area. (TIFF) [file pone.0188064.s001.tiff]
